# Survival Machine Learning Methods Improve Prediction of Histologic Transformation in Follicular and Marginal Zone Lymphomas

**DOI:** 10.3390/cancers17182952

**Published:** 2025-09-09

**Authors:** Tong-Yoon Kim, Tae-Jung Kim, Eun Ji Han, Gi-June Min, Seok-Goo Cho, Seoree Kim, Jong Hyuk Lee, Byung-Su Kim, Joon Won Jeoung, Hye Sung Won, Youngwoo Jeon

**Affiliations:** 1 Department of Hematology, Yeouido St. Mary’s Hospital, College of Medicine, The Catholic University of Korea, Seoul 07345, Republic of Korea; tyk@catholic.ac.kr; 2 Lymphoma and Cell Therapy Research Center, Yeouido St. Mary’s Hospital, College of Medicine, The Catholic University of Korea, Seoul 07345, Republic of Korea; beichest@catholic.ac.kr (G.-J.M.); chosg@catholic.ac.kr (S.-G.C.); 3 Department of Hospital Pathology, Yeouido St. Mary’s Hospital, College of Medicine, The Catholic University of Korea, Seoul 07345, Republic of Korea; kimecho@catholic.ac.kr; 4 Division of Nuclear Medicine, Department of Radiology, Yeouido St. Mary’s Hospital, College of Medicine, The Catholic University of Korea, Seoul 07345, Republic of Korea; iwao@catholic.ac.kr; 5 Department of Hematology, Seoul St. Mary’s Hospital, College of Medicine, The Catholic University of Korea, Seoul 06591, Republic of Korea; 6 Department of Oncology, Bucheon St. Mary’s Hospital, College of Medicine, The Catholic University of Korea, Seoul 14647, Republic of Korea; seoreek@catholic.ac.kr; 7 Department of Hematology, Incheon St. Mary’s Hospital, College of Medicine, The Catholic University of Korea, Seoul 21431, Republic of Korea; jonglee@catholic.ac.kr; 8 Department of Hematology, Eunpyeong St. Mary’s Hospital, College of Medicine, The Catholic University of Korea, Seoul 03312, Republic of Korea; 9 Department of Oncology, Daejeon St. Mary’s Hospital, College of Medicine, The Catholic University of Korea, Seoul 34943, Republic of Korea; 10 Department of Oncology, Uijeongbu St. Mary’s Hospital, College of Medicine, The Catholic University of Korea, Seoul 11765, Republic of Korea; woncomet@catholic.ac.kr

**Keywords:** aggressive histologic transformation, follicular lymphoma, marginal zone lymphoma, low-grade B-cell lymphoma, survival machine learning

## Abstract

Background/Objectives: Follicular lymphoma (FL) and marginal zone lymphoma (MZL) are low-grade B-cell lymphomas (LGBCLs) with indolent clinical courses but a lifelong risk of histologic transformation (HT) to aggressive lymphomas, particularly diffuse large B-cell lymphoma. Predicting HT can be challenging due to class imbalances and the inherent complexity of time-dependent events. While there are current prognostic indices for survival, they do not specifically address HT risk. This study aimed to develop and validate survival-based and traditional classification machine-learning models to predict HT in cohorts. Methods: Using a multicenter retrospective dataset (*n* = 1068), survival models (Cox proportional hazards, Lasso-Cox, Random Survival Forest, Gradient-boosted Cox [GBM-Cox], eXtreme Gradient Boosting [XGBoost]-Cox), and classification models (Logistic regression, Lasso logistic, Random Forest, Gradient Boosting, XGBoost) were compared. The best-performing survival models—XGBoost-Cox, Lasso-Cox, and GBM-Cox—were assessed on an independent test set (*n* = 92). Model sensitivity was maximized using optimal binary risk cutoff points based on Youden’s index. Results: Survival models showed superior predictive performance than classical classifiers, with XGBoost-Cox exhibiting the highest mean accuracy (85.3%), time-dependent area under the curve (0.795), sensitivity (98%), specificity (83.9%), and concordance index (0.836). Incorporating next-generation sequencing (NGS) data improved model accuracy and specificity, indicating that genetic factors improve HT prediction. Principal component analysis revealed distinct gene mutation patterns associated with HT risk, highlighting DNA-repair genes such as *TP53*, *BLM*, and *RAD50*. Conclusions: This study highlights the clinical value of survival-based machine-learning methods integrated with NGS data to personalize HT risk stratification for patients with FL and MZL.

## Simple Summary

Follicular lymphoma and marginal zone lymphoma are slow-growing non-Hodgkin lymphomas that can sometimes change into a more aggressive type called diffuse large B-cell lymphoma. This change, known as histologic transformation, is linked to worse outcomes, but it is difficult to predict in advance. We used patient data from seven hospitals to train and compare machine-learning models that estimate a risk of transformation. We found that models designed for survival analysis, especially those using advanced machine-learning methods, predicted transformation more accurately than traditional approaches. Adding genetic information further improved accuracy and helped identify important mutations linked to higher risk. These findings could help doctors personalize follow-up schedules and treatments, focusing attention on patients most likely to develop aggressive disease.

## 1. Introduction

Follicular lymphoma (FL) and marginal zone lymphoma (MZL) are types of indolent low-grade B-cell lymphomas (LGBCLs) that constitute a heterogeneous subset of non-Hodgkin lymphomas (NHLs). FL accounts for 12–25% of all NHL cases, whereas MZL, including mucosa-associated lymphoid tissue and nodal subtypes, accounts for 7–10.3% [1,2,3]. Despite their typically indolent clinical course, these lymphomas carry a persistent risk of histologic transformation (HT), often to aggressive lymphoma types such as diffuse large B-cell lymphoma. HT, with a reported incidence of 4–14.3%, is associated with significantly worse clinical outcomes [4,5].

Currently, established prognostic indices for LGBCLs include the Follicular Lymphoma International Prognostic Index (FLIPI) [6], PRIMA-Prognostic Index [7], and Mucosa-Associated Lymphoid Tissue Lymphoma Prognostic Index (MALT-IPI) [8]. However, these indices and next-generation sequencing (NGS)-enhanced models are tailored primarily toward survival prediction rather than identifying the specific risk of HT [9,10].

Predicting HT is challenging for several reasons. First, the relatively rare incidence of HT leads to significant class imbalance, which can bias algorithms toward the majority non-transformed group, confound performance metrics, and reduce statistical power. Second, HT is inherently time-dependent, rendering models based solely on fixed timepoints potentially misleading. Moreover, survival analysis requires careful consideration of right-censoring and time-varying hazard rates. 

Consequently, recent studies have attempted to improve HT prediction in LGBCLs by combining clinical parameters from established prognostic indices, such as FLIPI, with genetic data from NGS [11,12]. However, comprehensive comparisons between machine-learning survival models and classical classifiers are limited. Furthermore, the incremental predictive value of incorporating NGS data into these models is not yet fully understood.

To address this knowledge gap, this study developed and compared several predictive models to distinguish patients who experience HT from those who do not, employing both traditional classification methods and advanced survival-based machine-learning techniques. The study aimed to evaluate the performance of these models across independent FL and MZL cohorts, assess the added value of incorporating NGS data, and provide practical insights into optimizing HT risk prediction strategies.

## 2. Materials and Methods

### 2.1. Patient Selection

This multicenter, retrospective cohort study analyzed clinical data from 1068 patients diagnosed with LGBCLs at seven hospitals in the Republic of Korea: Yeouido, Seoul, Bucheon, Incheon, Daejeon, Eunpyeong, and Uijeongbu St. Mary’s. Patients diagnosed between January 2011 and February 2025 were included. Patients aged 18 years or older with a diagnosis of FL (grades 1, 2, or 3A) or MZL—covering splenic, nodal, and extranodal mucosa-associated lymphoid tissue (MALT) variants—were eligible for inclusion. The baseline diagnostic workup comprised a complete blood count, measurement of serum lactate dehydrogenase (LDH), bone marrow biopsy, and computed tomography scans of the neck, chest, abdomen, and pelvis.

The study was approved by the Institutional Review Board and Ethics Committee of the Catholic Medical Center, South Korea (approval numbers: SC23WISI0093 for the training/validation cohorts and XC25RIDI0050 for the test cohort). Owing to the retrospective nature of the analysis, the requirement for written informed consent was waived. All procedures were conducted in accordance with applicable ethical guidelines and regulations, including adherence to the principles outlined in the Declaration of Helsinki.

Demographic, clinical, and pathological data were systematically collected from medical records, including variables from established prognostic indices, the FLIPI and the MALT-IPI. Collected variables included demographic details (age at diagnosis and sex), clinical features (nodal involvement, Ann Arbor staging, pathological subtype, bone marrow involvement, and pleural effusion), and laboratory results (LDH and hemoglobin levels). Staging was consistently evaluated using the Lugano classification to maintain uniformity across participating institutions [13].

### 2.2. NGS

NGS was performed using the QIAseq Pan-cancer Multimodal Panel (Qiagen, Hilden, Germany), which targets DNA alterations across 523 cancer-related genes. DNA was isolated from formalin-fixed, paraffin-embedded (FFPE) tissue samples at diagnosis using the QIAamp DNA FFPE Tissue Kit (Qiagen, Hilden, Germany) in accordance with the manufacturer’s instructions. DNA quantity and quality assessments were conducted using the QIAseq DNA QuantiMIZE Array Kit (Qiagen, Hilden, Germany), ensuring optimal conditions for subsequent library preparation.

NGS library preparation adhered to the QIAseq Multimodal Panel HT Handbook (Qiagen). The workflow involved a series of steps, including enzymatic DNA fragmentation, end repair, addition of adenine overhangs (A-tailing), adapter ligation, and amplification via polymerase chain reaction (PCR). Unique molecular indices were incorporated during library construction to minimize PCR duplicates and sequencing artifacts, enhancing variant detection accuracy. Libraries were individually indexed, quantified using quantitative PCR, and assessed for quality using an Agilent Bioanalyzer to verify fragment size distribution and adapter removal.

Sequencing was performed on an Illumina platform (Illumina, San Diego, CA, USA) using Qiagen’s custom sequencing primers. Sequence data analysis involved variant calling using QIAGEN’s CLC Genomics Workbench v25.0 (QIAGEN Aarhus A/S, Aarhus, Denmark) software, with subsequent variant annotation and clinical interpretation using QIAGEN Clinical Insight Interpret v9.3.2.

### 2.3. Statistical Analysis

Categorical variables were compared using the Chi-square test or Fisher’s exact test, as appropriate. Overall survival (OS) was estimated using the Kaplan–Meier method, and differences between groups were assessed with the log-rank test. Univariate Cox proportional hazards models were used to identify predictors significantly associated with histologic transformation (HT). The cumulative incidence of HT was calculated using the Gray test, accounting for death as a competing risk. Model discrimination was assessed using Harrell’s concordance index (C-index), where values closer to 1.0 indicated stronger predictive ability, and values near 0.5 suggested no better than chance. Statistical significance was defined as a two-sided *p*-value of less than 0.05 [14].

All statistical analyses and data visualizations were performed using R software (version 4.2.3; R Foundation for Statistical Computing, Vienna, Austria). Survival models (Cox proportional hazards, Lasso-Cox [15], random survival forest [RSF] [16], gradient-boosted Cox [GBM-Cox] [17], and extreme gradient boosting [XGBoost]-Cox [18]) and classification algorithms (logistic regression, Lasso logistic regression [19], random forest [20], gradient boosting [21], and XGBoost [22]) were trained using a cohort comprising 592 patients (training set) and validated in an independent cohort of 384 patients (validation set) to predict HT. The top-performing survival models—XGBoost-Cox, Lasso-Cox, and GBM-Cox—were further evaluated on a separate test set (*n* = 92). Model performance was evaluated based on accuracy, the time-dependent area under the curve (AUC), sensitivity, and specificity at 6-, 12-, 18-, 24-, and 36-month intervals following diagnosis. The optimal binary risk cutoff values were determined by maximizing Youden’s index. Additional analyses evaluated the incremental improvement in predictive performance achieved by incorporating NGS-derived variables. NGS models were defined as those in which five or more mutations were detected in the test data. Principal component analysis (PCA) biplots were used to elucidate the contributions of NGS-derived predictors in the final predictive models. Nomograms were generated using the “rms” package to illustrate the impact of the variables in the models [23].

## 3. Results

### 3.1. Patient Characteristics and Survival Outcomes

Table 1 summarizes the baseline characteristics of the total cohort comprising 1068 patients. The median age at diagnosis was 52 years (range, 18–94 years). FL was the predominant subtype (*n* = 744, 69.7%), and MZL accounted for 324 cases (30.3%). In the total cohort, the HT group had a significantly higher prevalence of pleural effusion, elevated LDH levels, anemia, and Ann Arbor Stage III–IV disease (Table 1).

Patients diagnosed before 1 May 2020 were assigned to the training cohort (*n* = 592, 55.4%), whereas those diagnosed thereafter were placed in the validation/test cohort (*n* = 476, 44.6%). Comparisons between the training and validation/test cohorts indicated significant differences in several clinical features. Specifically, the validation/test cohort included a higher proportion of patients with involvement of more than four nodal sites, axial bone involvement, splenomegaly, anemia, and advanced-stage disease. In contrast, the training cohort had a significantly higher proportion of males and patients diagnosed with the MZL subtype (Table S1). However, subgroup analyses comparing the presence of HT between the training and validation/test cohorts revealed no significant differences across variables (Table S2).

**Figure 1:**
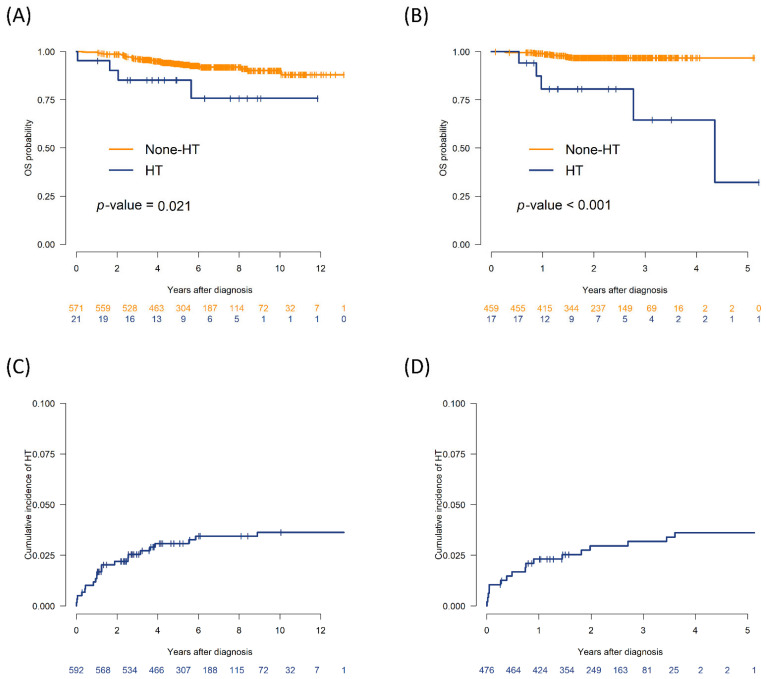
Overall survival and cumulative incidence of histologic transformation (HT) in the training and validation cohorts. (**A**) Kaplan–Meier curves for overall survival (OS) in the training set (*n* = 592), stratified by HT status. (**B**) OS curves in the validation/test set (*n* = 92), comparing patients with and without HT. (**C**) Cumulative incidence (CI) of HT in the training set, accounting for death as a competing risk. (**D**) CI of HT in the validation/test set, with death as a competing risk.

The median follow-up duration was 5.2 years (range, 1–13.1 years) in the training cohort and 2.2 years (range, 0.8–5.2 years) in the validation/test cohort. In the training cohort, the 5-year OS rate among patients who experienced HT was 85.2% (95% confidence interval [CI], 71.1–100%), which was significantly lower than the 93.7% (95% CI, 91.6–95.8%) observed in the non-HT group (*p* = 0.021). In the validation/test cohort, the 2-year OS rate was significantly lower in the HT group (80.7%; 95% CI, 63.2–100%) than in the non-HT group (96.8%; 95% CI, 95.1–98.5%) (*p* < 0.001; Figure 1A,B). The cumulative incidence of HT was 3.1% at 5 years in the training cohort and 3.0% at 2 years in the validation/test cohort (Figure 1C,D).

### 3.2. Model Comparison: Classification vs. Survival Models

We evaluated several machine-learning methods for predicting HT, including traditional classification models (logistic regression, Lasso regression, random forest, gradient boosting, and XGBoost) and survival-based models (Cox proportional hazards regression, Lasso-Cox, RSF, GBM-Cox, and XGBoost-Cox). Using the training and validation datasets, time-dependent AUCs were comparable between logistic regression and Cox proportional hazards models and between random forest and RSF. However, survival models employing regularization and boosting (Lasso-Cox, GBM-Cox, and XGBoost-Cox) demonstrated superior performance than their traditional classification counterparts (Lasso, GBM, and XGB).

**Figure 2:**
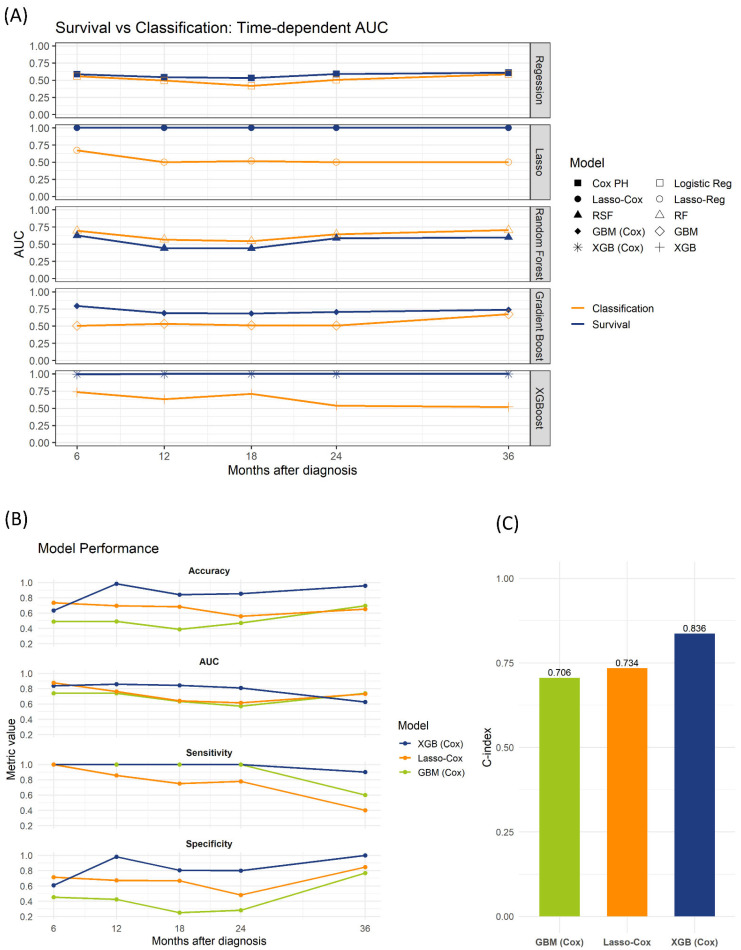
Time-dependent AUC in the training/validation cohorts and performance on the test set. (**A**) Time-dependent AUC for five landmark times (6, 12, 18, 24, and 36 months), comparing survival-based and classification-based models in the training and validation cohorts. Blue lines denote survival-based models; orange lines denote classification-based models. (**B**) Test set performance (*n* = 92) over the same timepoints for the three top survival models (XGBoost-Cox, Lasso-Cox, and GBM-Cox), showing accuracy, time-dependent AUC, sensitivity, and specificity. (**C**) Concordance indices (C-index) for GBM-Cox, Lasso-Cox, and XGBoost-Cox on the test set.

Further evaluation of the survival models on an independent test set (*n* = 92) included the optimization of binary risk thresholds using Youden’s index to improve sensitivity. The XGBoost-Cox model demonstrated the best performance, achieving the highest accuracy, time-dependent AUC, sensitivity, and specificity at 12-, 18-, and 24-month predictions. Additionally, XGBoost-Cox yielded the highest C-index (0.836), outperforming GBM-Cox (0.706) and Lasso-Cox (0.734) (Figure 2).

### 3.3. Incorporating NGS into the Model

**Figure 3:**
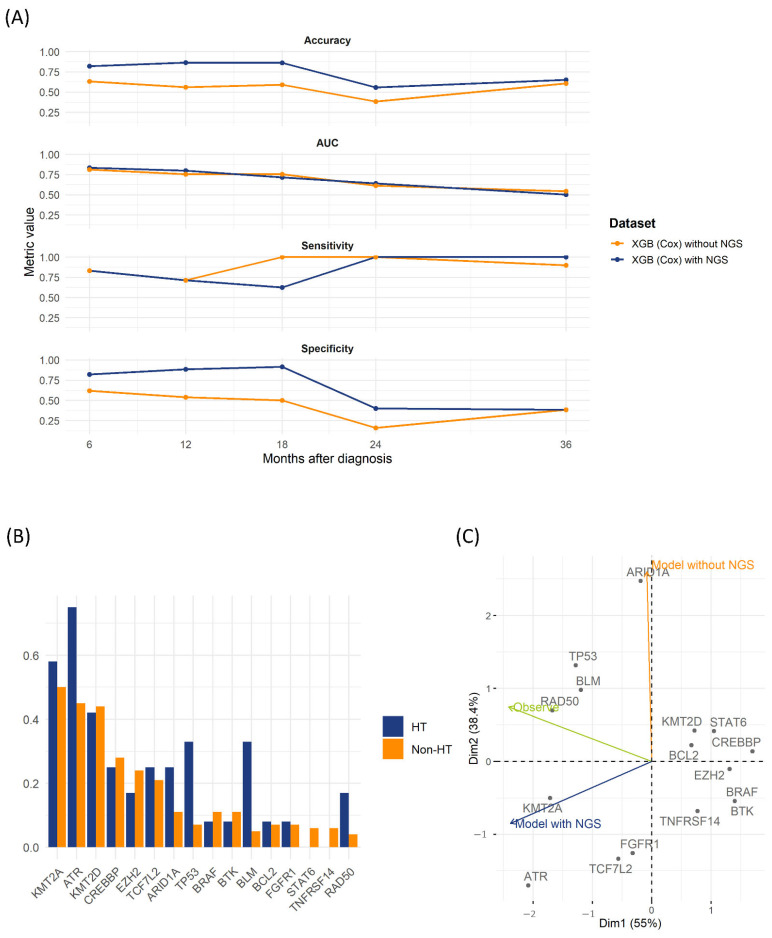
Test-set evaluation of XGBoost-Cox models and NGS predictor patterns. (**A**) Time-dependent performance of the XGBoost-Cox model with and without NGS at 6, 12, 18, 24, and 36 months for four metrics, showing accuracy, time-dependent AUC, sensitivity, and specificity. (**B**) Bar plots showing the prevalence of the top 15 NGS-derived predictor variables in patients who developed HT (blue) and those who did not (orange) in the test cohort (*n* = 92). (**C**) PCA biplot of the same 15 NGS predictors in the test set. Arrows indicate the projection directions of the model without NGS (orange), the model with NGS (navy), and the observed HT vector (green), showing alignment with actual transformation events. Gray dots correspond to individual gene loadings on Principal Components 1 (*x*-axis, 55 % variance) and 2 (*y*-axis, 38.4 % variance).

We further examined the impact of incorporating NGS data into predictive models using the test set. Cox proportional hazards regression identified significant hazard ratios for *TP53*, *KMT2A*, *BLM*, *ATR*, and *RAD50* gene alterations related to HT (Table 2). Incorporating NGS variables significantly improved the XGBoost-Cox model, consistently increasing accuracy and specificity across multiple timepoints. Although the increase in the AUC was modest, the integration of NGS data improved long-term accuracy compared with the clinical-only model (Figure 2A). *TP53* and *BLM* mutations were notably more prevalent in the HT group than in the non-HT group (Figure 3B and Table 2). The nomogram in Figure S2 illustrates the relative impact of variables according to the models.

PCA highlighted distinct patterns among predictors. The first two Principal Components (PC1 and PC2) explained 55% and 38.4% of the variance, respectively (Figure 3C). PC1 separated genes with strong positive loadings (*CREBBP*, *BCL2*, *STAT6*, *KMT2D*, *TNFRSF14*, *BTK*, *BRAF*, and *EZH2*) from negatively loaded NGS-driven alterations (*KMT2A*, *ATR*, *FGFR1*, and *TCF7L2*). The clinical-only model (excluding NGS data) primarily loaded on PC2, indicating reliance on clinically distinct features. In contrast, the NGS-integrated model demonstrated negative PC1 loading, closely clustering with key NGS mutations. The “Observe” vector, representing actual HT events, aligned closely with DNA-repair or tumor-suppressor genes, including *TP53*, *BLM*, and *RAD50*. These findings underline the clinical and prognostic importance of incorporating NGS-derived genetic profiles into HT-prediction models for patients with LGBCLs.

## 4. Discussion

This comprehensive study involving 1068 patients with LGBCLs showed that survival-based models significantly improved the prediction of HT. Time-dependent modeling emerged as particularly valuable, underscoring the importance of incorporating temporal factors into HT predictions. Our findings align closely with those of previous research including that by Ismael et al., which reported a C-index of 0.618 for Cox proportional hazards models based solely on clinical data. Similarly, the present study yielded a comparable C-index of approximately 0.586 [11]. 

Notably, certain survival modeling approaches had distinct advantages. Although traditional Cox proportional hazards models performed similarly to logistic regression, models employing regularization, such as Lasso-Cox, clearly outperformed their corresponding classical classifiers (e.g., Lasso regression). The inherent instability of coefficient estimation in the standard Cox proportional hazards model due to multicollinearity or limited sample sizes underscores the advantage of Lasso-Cox. Using the regularization parameter λ, Lasso-Cox reduces overfitting by selectively including relevant variables. This approach proves especially beneficial in high-dimensional, sparse settings where few predictors genuinely impact prognosis [15].

Additionally, GBM-Cox and XGBoost-Cox consistently demonstrated superior predictive performance than RSF models. This superiority is likely attributable to the fact that gradient boosting builds decision trees sequentially. Each tree specifically addresses errors from prior steps. This process increases the model’s ability to capture difficult-to-predict cases and helps reduce bias. In contrast, RSF averages fully grown trees using bagging, making it inherently less capable of identifying subtle or low-frequency mutations and complex interactions [24,25]. Both XGBoost and XGBoost-Cox utilize gradient boosting algorithms, but they differ distinctly in their respective loss functions. Zha et al. previously demonstrated that the FLIPI-C model, initially developed using XGBoost, could be improved by applying the XGBoost-Cox model [26]. In this study, we applied optimal binary risk thresholds derived by maximizing Youden’s index to enhance model sensitivity. Clinically, sensitivity is typically more crucial than specificity in predicting HT, as clinicians prioritize accurately identifying patients who may develop HT rather than identifying those who will not. By employing Youden’s index, our models could effectively address class imbalance, allowing customized optimization to better serve clinical decision-making.

Incorporating NGS data notably improved model accuracy by increasing specificity. Mutation analysis revealed that *TP53* mutations were significantly more prevalent in patients who experienced HT, aligning well with the existing literature [27,28]. Notably, observed HT events closely aligned with DNA-repair and tumor-suppressor genes, including *TP53*, *BLM*, and *RAD50*, suggesting their critical roles in transformation risk. However, discrepancies were noted regarding some mutations previously reported as significant. For instance, although previous studies associated mutations in *MYC*, *CDKN2A*, and *TNFRSF14* with HT, our cohort confirmed only *TP53* mutations to be significantly associated with HT [11]. Although BLM mutations were prevalent in HT cases in our data, direct clinical evidence remains limited. Existing support is primarily derived from mouse model studies. Similarly, *RAD50* mutation and *KMT2A* rearrangement were associated with higher hazard ratios in our cohort. However, prior clinical documentation of their specific roles in HT remains sparse. This discrepancy indicates potential differences arising from variations in lymphoma subtypes or ethnic diversity within cohorts [29,30,31]. 

Our findings emphasize the importance of integrating advanced machine-learning methods into clinical practice, particularly in cohorts with inherent limitations such as class imbalance or variable-specific sparsity. Compared with classical classification methods, survival-based machine learning, especially XGBoost-Cox, demonstrated superior predictive capabilities for HT in patients with FL or MZL. The nonlinear, interaction-sensitive nature of tree-based models captures intricate relationships among NGS variables more effectively than linear approaches, thus improving predictive accuracy.

Nevertheless, there are notable limitations regarding the interpretability and direct clinical applicability of machine-learning models. The optimal timing for evaluating patient risks of HT remains a significant consideration. The median transformation period in our cohort was approximately 1.7 years, slightly shorter than the previously reported interval of approximately 2 years, which is associated with a high incidence of HT detection [32]. Thus, clinicians should particularly monitor early-stage risk factors, including advanced disease stage, elevated LDH levels, and anemia, during this critical period [4,5].

This study also has inherent limitations related to its retrospective design and the absence of external validation, especially for models incorporating NGS data. Prospective validation, including assessments involving circulating tumor DNA and multimodal imaging modalities, is recommended to enhance and refine predictive models for HT. Additionally, the lack of standardized treatment-related outcome measures represents another limitation. Variations in treatment timing and decisions across different institutions render such data unsuitable for incorporation into predictive models. Future research should therefore integrate treatment data into time-dependent models. Lastly, the relatively small number of HT cases posed challenges. Nonetheless, our analysis reflects real-world clinical scenarios characterized by inherent class imbalances.

## 5. Conclusions

This multicenter retrospective study demonstrated that survival-based machine-learning models, particularly XGBoost-Cox, significantly enhanced the prediction of HT in patients with FL and MZL. Incorporating NGS data markedly improved model accuracy, highlighting key genetic alterations such as *TP53*, *BLM*, and *RAD50* as significant predictors. These findings emphasize the clinical value of integrating molecular profiling into routine prognostic assessments to enable early identification and timely intervention for patients at high risk of HT. Future prospective studies and external validation are required to confirm these findings and facilitate the development of personalized therapeutic strategies for patients with LGBCLs.

## Figures and Tables

**Table 1:** Characteristics of patients in the total cohort.

	Total	Non-HT	HT	*p*
	(*n* = 1068)	(*n* = 1030)	(*n* = 38)	
Age >60 years, *n* (%)	317 (29.7)	302 (29.3)	15 (39.5)	0.244
Sex				0.396
Female	564 (52.8)	547 (53.1)	17 (44.7)	
Male	504 (47.2)	483 (46.9)	21 (55.3)	
Diagnosis subtype, *n* (%)				0.286
FL	744 (69.7)	721 (70.0)	23 (60.5)	
MZL	324 (30.3)	309 (30.0)	15 (39.5)	
Involvement >4 nodal sites, *n* (%)	288 (27.0)	274 (26.6)	14 (36.8)	0.226
Axial bone involvement, *n* (%)				0.504
Absent	741 (69.4)	717 (69.6)	24 (63.2)	
Present	327 (30.6)	313 (30.4)	14 (36.8)	
Spleen involvement, *n* (%)				0.101
Absent	902 (84.5)	874 (84.9)	28 (73.7)	
Present	166 (15.5)	156 (15.1)	10 (26.3)	
Pleural effusion, *n* (%)				0.001
Absent	1025 (96.0)	993 (96.4)	32 (84.2)	
Present	43 (4.0)	37 (3.6)	6 (15.8)	
LDH elevation, *n* (%)	186 (17.4)	174 (16.9)	12 (31.6)	0.033
Hemoglobin, *n* (%)				<0.001
≥12 g/dL	924 (86.5)	901 (87.5)	23 (60.5)	
<12 g/dL	144 (13.5)	129 (12.5)	15 (39.5)	
Ann Arbor Stage *n* (%)				0.011
I–II	422 (39.5)	415 (40.3)	7 (18.4)	
III–IV	646 (60.5)	615 (59.7)	31 (81.6)	

*n*, number; *p*, *p*-value; HT, aggressive histologic transformation; FL, follicular lymphoma; MZL, marginal zone lymphoma; LDH, lactate dehydrogenase.

**Table 2:** Mutation profiles and results of univariate analyses in the NGS cohort.

	Non-HT	HT	*p*	HR (95% CI)	*p*
	(*n* = 80)	(*n* = 12)			
*KMT2D*			>0.999		0.488
Wild	45 (56.2)	7 (58.3)		Reference	
Mutation	35 (43.8)	5 (41.7)		1.56 (0.45, 5.43)	
*CREBBP*			>0.999		0.807
Wild	58 (72.5)	9 (75.0)		Reference	
Mutation	22 (27.5)	3 (25.0)		0.85 (0.23, 3.14)	
*TP53*			0.029		0.006
Wild	74 (92.5)	8 (66.7)		Reference	
Mutation	6 (7.5)	4 (33.3)		6.13 (1.70, 22.1)	
*ARID1A*			0.39		0.105
Wild	71 (88.8)	9 (75.0)		3.08 (0.79, 12.0)	
Mutation	9 (11.2)	3 (25.0)		Reference	
*STAT6*			0.835		0.998
Wild	75 (93.8)	12 (100.0)		Reference	
Mutation	5 (6.2)	0 (0.0)		Not applicable	
*TNFRSF14*			0.835		0.998
Wild	75 (93.8)	12 (100.0)		Reference	
Mutation	5 (6.2)	0 (0.0)		Not applicable	
*BCL2*			>0.999		0.994
Wild	74 (92.5)	11 (91.7)		Reference	
Mutation	6 (7.5)	1 (8.3)		1.01 (0.12, 8.14)	
*EZH2*			0.86		0.504
Wild	61 (76.2)	10 (83.3)		Reference	
Mutation	19 (23.8)	2 (16.7)		0.59 (0.13, 2.77)	
*BRAF*			>0.999		0.402
Wild	71 (88.8)	11 (91.7)		Reference	
Mutation	9 (11.2)	1 (8.3)		0.4 (0.05, 3.36)	
*KMT2A*			0.819		0.031
Wild	40 (50.0)	5 (41.7)		Reference	
Rearrangement	40 (50.0)	7 (58.3)		5.46 (1.16, 25.6)	
*BLM*			0.007		0.001
Wild	76 (95.0)	8 (66.7)		Reference	
Mutation	4 (5.0)	4 (33.3)		8.44 (2.36, 30.1)	
*BTK*			>0.999		0.402
Wild	71 (88.8)	11 (91.7)		Reference	
Mutation	9 (11.2)	1 (8.3)		0.4 (0.05, 3.36)	
*FGFR1*			>0.999		0.451
Wild	74 (92.5)	11 (91.7)		Reference	
Rearrangement	6 (7.5)	1 (8.3)		2.26 (0.27, 18.8)	
*ATR*			0.103		0.007
Wild	44 (55.0)	3 (25.0)		Reference	
Mutation	36 (45.0)	9 (75.0)		6.86 (1.68, 27.9)	
*TCF7L2*			>0.999		0.226
Wild	63 (78.8)	9 (75.0)		Reference	
Mutation	17 (21.2)	3 (25.0)		2.44 (0.58, 10.3)	
*RAD50*			0.247		0.011
Wild	77 (96.2)	10 (83.3)		Reference	
Mutation	3 (3.8)	2 (16.7)		8.06 (1.61, 40.3)	

CI, confidence interval; HR, hazard ratio; HT, aggressive histologic transformation.

## Data Availability

The original data presented in the study are openly available in Dryad (http://datadryad.org/share/jP9OP57cXRLW7NmXwu9OOrdbvYJ6ySgAyQB5pXAhvV4, accessed on 7 September 2025).

## Supplementary Materials

The following supporting information can be downloaded at https://www.mdpi.com/article/10.3390/cancers17182952/s1, Table S1: Characteristic of patients with train/validation versus test set; Table S2: Characteristic of patients with histologic transformation in train/validation versus test set; Figure S1: Flow diagram of patient selection and cohort stratification; Figure S2: Nomogram for aggressive histologic transformation. (A) In the train set and (B) in the test set with next-generation sequencing.

## Abbreviations

The following abbreviations are used in this manuscript:

AUC	Area under the curve
DLBCL	Diffuse large B-cell lymphoma
FL	Follicular lymphoma
FLIPI	Follicular Lymphoma International Prognostic Index
GBM-Cox	Gradient-boosted Cox model
HT	Histologic transformation
LGBCL	Low-grade B-cell lymphoma
MALT-IPI	Mucosa-Associated Lymphoid Tissue Lymphoma Prognostic Index
ML	Machine learning
MZL	Marginal zone lymphoma
NGS	Next-generation sequencing
NHL	Non-Hodgkin lymphoma
PCA	Principal component analysis
RSF	Random Survival Forest
CI	Confidence interval
HR	Hazard ratio
FFPE	Formalin-fixed, paraffin-embedded
