# Supplementary material for: Survival Machine Learning Methods Improve Prediction of Histologic Transformation in Follicular and Marginal Zone Lymphomas
Source: Cancers (Basel). 2025 Sep 9;17(18):2952. doi: 10.3390/cancers17182952 (PMC12468402; doi:10.3390/cancers17182952)
Supplement: Supplementary file 1 [file cancers-17-02952-s001.zip › cancers-3841807-supplementary.pdf]

Supplementary Materials

Table S1. Characteristic of patients with train/validation versus test set.

**Cohort**	**Total (N=1068)**	**Train set (N=592)**	**Validation/ Test set(N=476)**	***P***
Histologic transformation, N (%)				>0.999
Non-HT	1030 (96.4)	571 (96.5)	459 (96.4)	
HT	38 (3.6)	21 (3.5)	17 (3.6)	
Age >60 years, N (%)	317 (29.7)	175 (29.6)	142 (29.8)	0.977
Sex				0.018
Female	564 (52.8)	293 (49.5)	271 (56.9)	
Male	504 (47.2)	299 (50.5)	205 (43.1)	
Diagnosis subtype, N (%)				<0.001
FL	744 (69.7)	353 (59.6)	391 (82.1)	
MZL	324 (30.3)	239 (40.4)	85 (17.9)	
Involvement >4 nodal sites, N (%)	288 (27.0)	130 (22.0)	158 (33.2)	<0.001
Axial bone involvement, N (%)				0.002
Absent	741 (69.4)	434 (73.3)	307 (64.5)	
Present	327 (30.6)	158 (26.7)	169 (35.5)	
Spleen involvement, N (%)				<0.001
Absent	902 (84.5)	523 (88.3)	379 (79.6)	
Present	166 (15.5)	69 (11.7)	97 (20.4)	
Pleural effusion, N (%)				0.175
Absent	1025 (96.0)	573 (96.8)	452 (95.0)	
Present	43 (4.0)	19 (3.2)	24 (5.0)	
LDH elevation, N (%)	186 (17.4)	110 (18.6)	76 (16.0)	0.299
Hemoglobin, N (%)				0.041
≥12 g/dL	924 (86.5)	524 (88.5)	400 (84.0)	
<12 g/dL	144 (13.5)	68 (11.5)	76 (16.0)	
Ann Arbor Stage N (%)				<0.001
I-II	422 (39.5)	293 (49.5)	129 (27.1)	
III–IV	646 (60.5)	299 (50.5)	347 (72.9)	

N, number; P, p-value; HT, aggressive histologic transformation; FL, follicular lymphoma; MZL, marginal zone lymphoma; LDH, lactate dehydrogenase.

Table S2. Characteristic of patients with histologic transformation in train/validation versus test set.

	**Total**	**Train set / Validation**	**Test set**	***P***
	(N=38)	(N=21)	(N=17)	
Age >60 years, N (%)	15 (39.5)	9 (42.9)	6 (35.3)	0.888
Sex				0.945
Female	17 (44.7)	10 (47.6)	7 (41.2)	
Male	21 (55.3)	11 (52.4)	10 (58.8)	
Diagnosis subtype, N (%)				0.14
- FL	23 (60.5)	10 (47.6)	13 (76.5)	
- MZL	15 (39.5)	11 (52.4)	4 (23.5)	
Involvement >4 nodal sites, N (%)	14 (36.8)	8 (38.1)	6 (35.3)	>0.999
Axial bone involvement, N (%)				0.606
Absent	24 (63.2)	12 (57.1)	12 (70.6)	
Present	14 (36.8)	9 (42.9)	5 (29.4)	
Spleen involvement, N (%)				>0.999
Absent	28 (73.7)	15 (71.4)	13 (76.5)	
Present	10 (26.3)	6 (28.6)	4 (23.5)	
Pleural effusion, N (%)				>0.999
Absent	32 (84.2)	18 (85.7)	14 (82.4)	
Present	6 (15.8)	3 (14.3)	3 (17.6)	
LDH elevation, N (%)	12 (31.6)	8 (38.1)	4 (23.5)	0.542
Hemoglobin, N (%)				>0.999
≥12 g/dL	23 (60.5)	13 (61.9)	10 (58.8)	
<12 g/dL	15 (39.5)	8 (38.1)	7 (41.2)	
Ann Arbor Stage N (%)				0.17
I-II	7 (18.4)	6 (28.6)	1 (5.9)	
III–IV	31 (81.6)	15 (71.4)	16 (94.1)	

N, number; P, p-value; HT, aggressive histologic transformation; FL, follicular lymphoma; MZL, marginal zone lymphoma; LDH, lactate dehydrogenase.

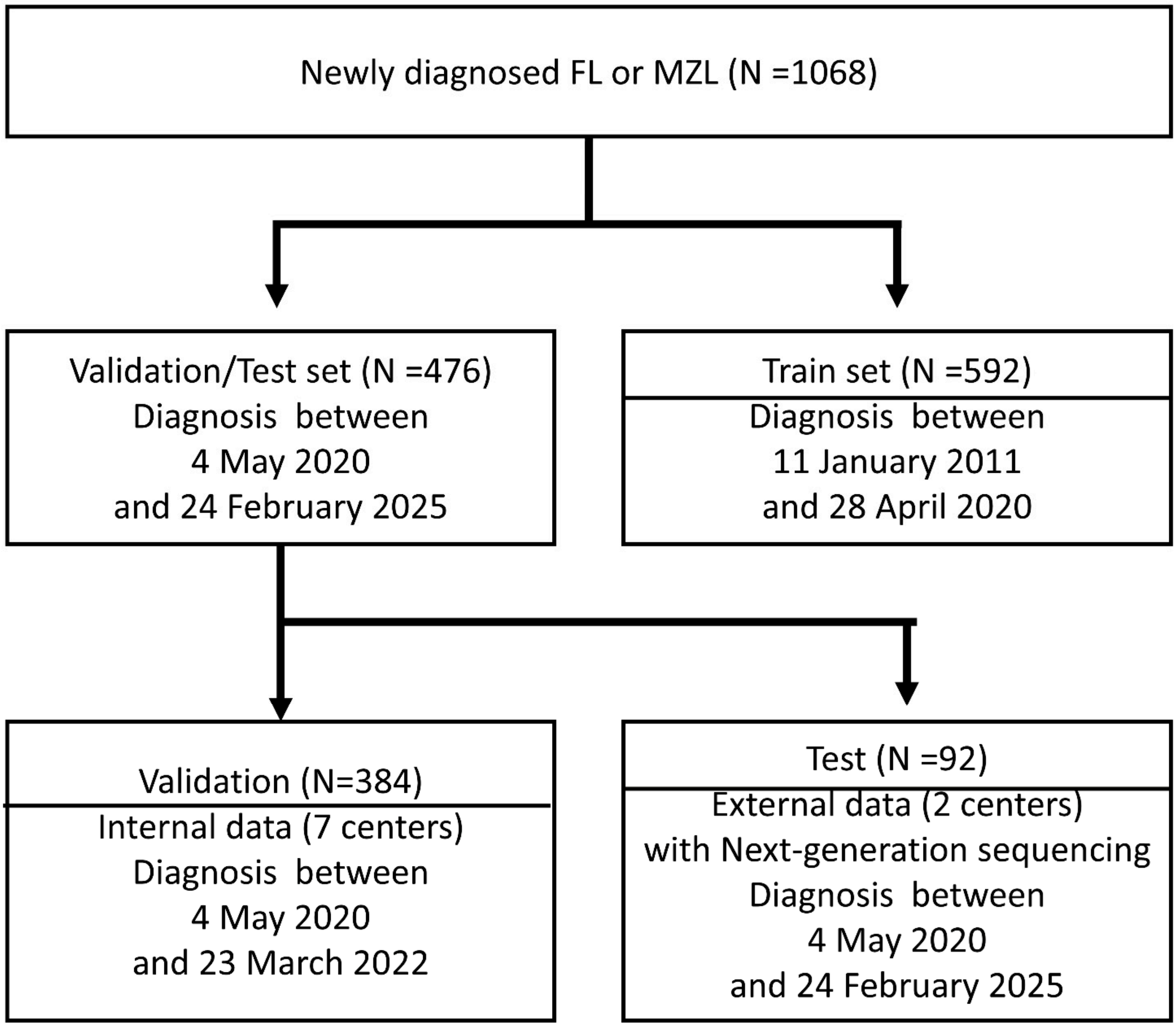

Figure S1. Flow diagram of patient selection and cohort stratification FL, follicular lymphoma; MZL, marginal zone lymphoma.

(A)

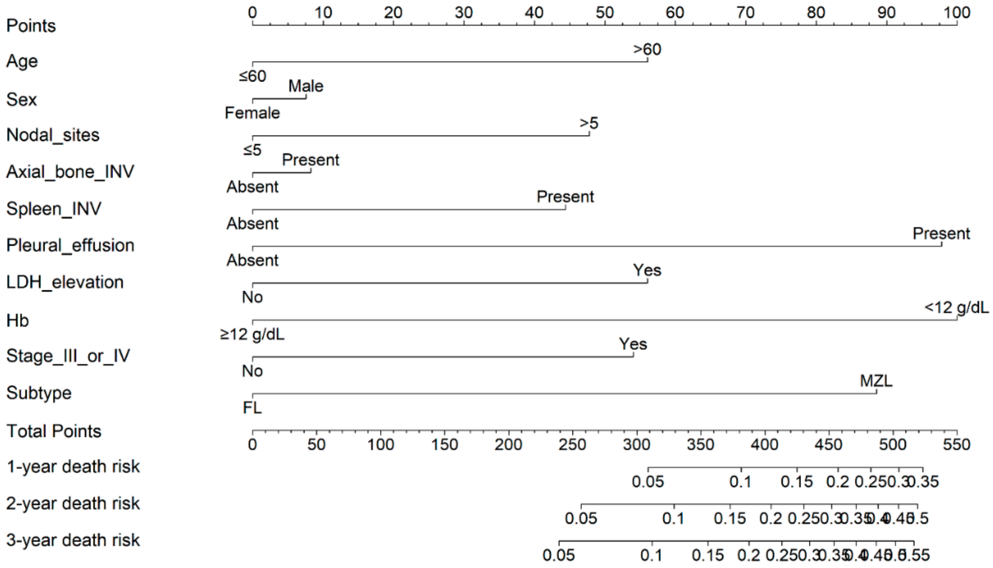

Points
Age
Sex
Nodal_sites
Axial_bone_INV
Spleen_INV
Pleural_effusion
LDH_elevation
Hb
Stage_III_or_IV
Subtype
Total Points
1-year death risk
2-year death risk
3-year death risk
0 10 20 30 40 50 60 70 80 90 100
>60
≤60
Male
Female
>5
≤5
Present
Absent
Present
Absent
Present
Absent
Yes
No
<12 g/dL
≥12 g/dL
Yes
No
MZL
FL
0 50 100 150 200 250 300 350 400 450 500 550

(B)

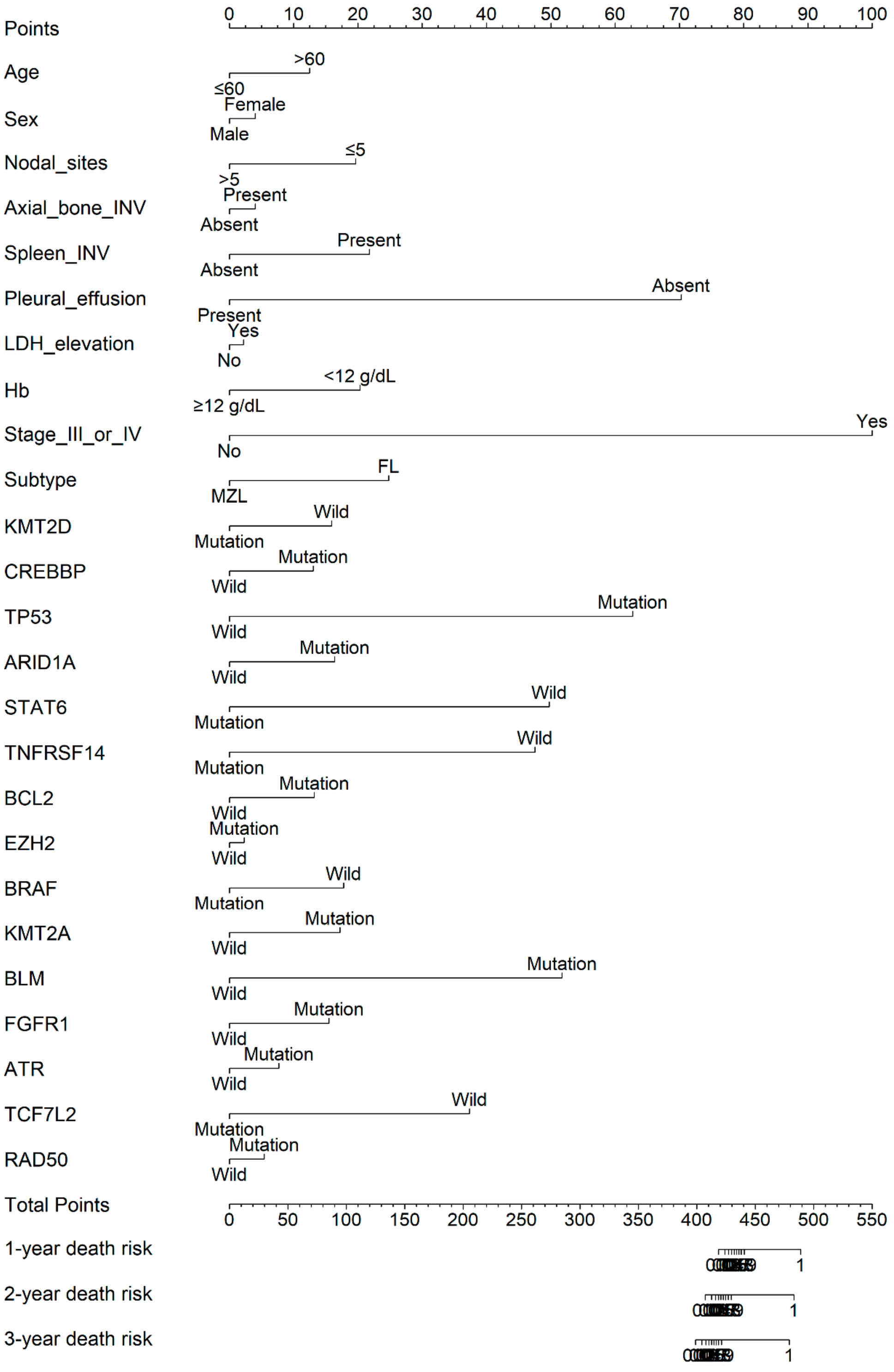

Figure S2. Nomogram for aggressive histologic transformation. (A) In the train set and (B) in the test set with next-generation sequencing.
